# Non-compliance as ethical dilemma for kidney transplantation

**DOI:** 10.1192/j.eurpsy.2024.1200

**Published:** 2024-08-27

**Authors:** L. Rossini Gajsak, L. Zibar

**Affiliations:** ^1^Department of Integrative Psychiatry, University Psychiatric Hospital Sveti Ivan; ^2^Department for Nephrology, Internal Clinic, Clinical Hospital Merkur, Zagreb; ^3^Faculty of Medicine, Department for Pathophysiology, University Juraj Strossmayer, Osijek, Croatia

## Abstract

**Introduction:**

Allocating a kidney transplant to a non-compliant recipient could present a triple damage: to the donor (and family of a deceased donor), for the recipient (who will experience rejection) and for another potential recipient on the waiting list (who missed the chance for the transplant). Having in mind that kidney transplantation (TX) is the best choice of renal replacement therapy, a thorough individual endeavor to predict the outcome of a TX in a non-compliant candidate is necessary to avoid a worse option. Non-compliance could origin from maladaptation, psychological limitations or a psychiatric condition.

**Objectives:**

Here we present a 46 years old male patient on chronic hemodialysis (HD) for 4 years due to end stage diabetic kidney disease. He is extremely non-adherent to HD related recommendations, occasionally skipping the sessions, gaining up to 10 kg weight overload between the sessions and avoided visiting psychiatrist, so far. Our objectives were to explore the presence and severity of non-compliance as ethical dilemma for kidney transplantation.

**Methods:**

Reviewing the patient’s medical data.

**Results:**

Unlike to non-obedience to dietary and behavioral medical advice, this patient is very much adherent to pharmacological medication. Staying on HD he is constantly on the edge of vital danger, risking pulmonary edema or hyperkalemia related cardiac events. The most important compliance in a kidney transplant patient is adherence to immunosuppressive therapy. In this particular patient we could predict adherence to immunosuppressive medication after a TX and getting rid of volume overload and hyperkalemia once restoring kidney transplant function.

**Conclusions:**

Pretransplant non-compliance in kidney transplant candidate is not always an obstacle for kidney TX. In some cases, as in the one here described, a TX is better option than staying on HD, avoiding the previously described triple ethical damage - to the donor, the recipient and patients waiting on list, while we could predict a good outcome of the TX. Including psychiatrist into the work up and management should not be skipped.

**Disclosure of Interest:**

None Declared

